# Targeted DNA vaccines for enhanced induction of idiotype-specific B and T cells

**DOI:** 10.3389/fonc.2012.00154

**Published:** 2012-10-30

**Authors:** Agnete B. Fredriksen, Inger Sandlie, Bjarne Bogen

**Affiliations:** Centre for Immune Regulation, Institute of Immunology, University of Oslo and Oslo University HospitalOslo, Norway

**Keywords:** idiotype, vaccine, lymphoma, multiple myeloma, antigen-presenting cells

## Abstract

**Background:** Idiotypes (Id) are antigenic determinants localized in variable (V) regions of Ig. Id-specific T and B cells (antibodies) play a role in immunotherapy of Id^+^ tumors. However, vaccine strategies that enhance Id-specific responses are needed. **Methods:** Id^+^ single-chain fragment variable (scFv) from multiple myelomas and B cell lymphomas were prepared in a fusion format that bivalently target surface molecules on antigen-presenting cells (APC). APC-specific targeting units were either scFv from APC-specific mAb (anti-MHC II, anti-CD40) or chemokines (MIP-1α, RANTES). Homodimeric Id-vaccines were injected intramuscularly or intradermally as plasmids in mice, combined with electroporation. **Results:** (i) Transfected cells secreted plasmid-encoded Id^+^ fusion proteins to extracellular fluid followed by binding of vaccine molecules to APC. (ii) Targeted vaccine molecules increased Id-specific B and T cell responses. (iii) Bivalency and xenogeneic sequences both contributed to enhanced responses. (iv) Targeted Id DNA vaccines induced tumor resistance against challenges with Id^+^ tumors. (v) Human MIP-1α targeting units enhanced Id-specific responses in mice, due to a cross reaction with murine chemokine receptors. Thus, targeted vaccines designed for humans can be quality tested in mice. (vi) Human Id^+^ scFv from four multiple myeloma patients were inserted into the vaccine format and were successfully tested in mice. (vii) Human MIP-1α vaccine proteins enhanced human T cell responses *in vitro.* (viii) A hypothetical model for how the APC-targeted vaccine molecules enhance Id-specific T and B cells is presented. **Conclusion:** Targeted DNA Id-vaccines show promising results in preclinical studies, paving the way for testing in patients.

## INDUCTION OF ANTI-Id ANTIBODIES TO SYNGENEIC Ig

In their classical studies, Oudin and Michel (1963) and Kunkel et al. (1963) immunized rabbits with human Ig and observed that the rabbits made antibodies specific for the injected Ig. The antigen recognized by the rabbit antibodies was called idiotype (Id). In hindsight, these observations are perhaps not so surprising since xenogeneic Ig variable (V) regions, where the Id is located, are expected to be immunogenic when crossing species barriers, due to inter species sequence differences between V regions.

Maybe more surprising, Eisen and colleagues demonstrated that immunization with a monoclonal Ig, myeloma protein M315, induced anti-Id antibodies even in syngeneic BALB/c mice (Sirisinha and Eisen, 1971). This important finding indicated that Ig V regions can be autoimmunogenic, as later formally demonstrated by Rodkey (1974). In order to elicit syngeneic anti-Id antibodies, Eisen and colleagues used an extensive immunization schedule, starting with M315 in complete Freund’s adjuvant. Thus, syngeneic Ig appeared to be weak autoantigens in terms of eliciting anti-Id antibodies (Sirisinha and Eisen, 1971). Moreover, immunogenicity of various monoclonal Ig differed. In particular, an abundant Ig with germline-encoded V regions (T15) failed to induce anti-Id antibodies, presumably due to self-tolerance (Sakato and Eisen, 1975). Hence, somatic mutations and/or V(D)J junctional diversity appear to be required for sufficient “foreignness” of Id to be immunogenic in an autologous setting. These seminal observations of Sirisinha and Eisen (1971) have later been repeated with a large number of Igs with essentially similar results.

## INDUCTION OF ANTI-Id ANTIBODIES IS DEPENDENT ON Id-SPECIFIC T CELLS

T cell deficient mice, either nude mice (Schrater et al., 1979) or neonatally thymectomized mice (Cosenza et al., 1977), do not produce anti-Id antibodies upon immunization with Ig. Thus, Ig is a T dependent antigen like most protein antigens. Consistent with this, Janeway et al. (1975) and Jorgensen and Hannestad (1977) found that immunization of mice with Ig induced Id-specific T cells that could help secondary hapten-specific B cells in adoptive cell transfer experiments performed essentially *ad modum* Mitchison (1971) and Rajewsky (1971). Jorgensen and Hannestad (1980), 1982 further found that elicitation of Id-specific T cells specific for the M315 monoclonal Ig was under the influence of MHC-linked immune response (Ir) genes. The Id determinant was localized to complementarity determining region (CDR) 3 of the λ2^315^ Ig light (L) chain (Jorgensen et al., 1983; Bogen et al., 1985). Bogen et al. (1986a),b and Bogen and Lambris (1989) cloned T cells specific for this particular Id (λ2^315^)-determinant and showed that these T cells were CD4^+^ and recognized aa 91–101 of the λ2^315^ L chain presented on the MHC class II molecule I-E^d^ in BALB/c mice. Moreover, it was demonstrated that Ig requires antigen processing by the antigen-presenting cells (APC) for the Id-peptide to be presented on MHC class II molecules (Weiss and Bogen, 1989, 1991). Id-specific TCR transgenic T cells of this particular specificity indeed help anti-Id B cells in the presence of Id^+^ Ig (Jacobsen et al., 2010).

## IDIOTYPIC NETWORK THEORY: ANTI-Id (Ab2) AS A MIMICK OF ANTIGEN, AND AS INHIBITOR OF PATHOGENIC AUTOANTIBODIES

The observation that Ig is autoimmunogenic paved the way for the idiotypic network theory of Jerne (1974). Despite considerable criticism of the network theory over the years, there is much evidence to support an influence of Id in immune regulation – even though the mechanisms may not be exactly as suggested by Jerne (1974). In particular, immunization with anti-Id (Ab2) antibodies has been shown to induce anti-anti-Id (Ab3) antibodies that can bind the “original” Ag, a feature they share with Id^+^ (Ab1) antibodies. Thus, anti-Id antibodies may function as a mimic of antigen. This principle may be of practical importance since antigens sometimes are poorly immunogenic, or difficult to obtain in sufficient amounts for immunization. In these cases, anti-Id monoclonal Ab (mAb) may be used as a surrogate antigen for vaccination purposes. This strategy has recently been successfully exploited by using anti-Id mAb as immunogen for induction of anti-anti-Id Ab that bind gangliosides on lung cancer (Hernandez et al., 2011) and melanoma (Ramos et al., 2011) cells. Another interesting function of anti-Id Ab is that they appear to block the function of pathogenic Id^+^ autoantibodies as suggested in studies in type I diabetes (Oak et al., 2008). In either case, technologies to increase immunogenicity of Ig V region antigenic determinants by immunization, as described herein, is warranted.

## IDIOTYPES AS TUMOR-SPECIFIC ANTIGENS

Another aspect of Id that has stood the test of time is their role as a target on malignant B cells for an immune attack. Lynch et al. (1972) found that when mice immunized with Id^+^ M315 myeloma protein were later challenged with the Id^+^ MOPC315 plasmacytoma tumor cells, the mice were protected against tumor development. Since plasmacytoma cells (an extramedullary form of multiple myeloma) secrete copious amounts of the Id^+^ myeloma protein, anti-Id antibodies should be blocked by omnipresent myeloma protein and appear not important for protection. Rather, Id-specific CD4^+^Th1 cells have been shown to kill the tumor cells by a mechanism that involves IFN γ and tumor infiltrating M1 macrophages (Lauritzsen et al., 1994; Dembic et al., 2000; Corthay et al., 2005, 2009; Haabeth et al., 2011). Immunotherapy with anti-Id antibodies may be more important in B lymphomas that secrete little Ig but that express high amounts of surface Ig as targets for the anti-Id antibodies. Indeed, anti-Id antibodies have a therapeutic effect against B lymphomas in experimental models (George et al., 1987; Kaminski et al., 1987). However, phase III trials in humans have been negative in two cases (Levy et al., 2008; Freedman et al., 2009), while in a third study an improvement of disease-free survival in patients vaccinated in first remission was observed (Schuster et al., 2011).

## IDIOTYPES ARE WEAK ANTIGENS: CONVENTIONAL AND NOVEL STRATEGIES TO INCREASE THEIR IMMUNOGENICITY

As reviewed above, there are two important reasons for efficient induction of anti-Id immunity: (i) use of anti-anti-Id mAb (Ab2) as mimic of antigen for vaccine purposes and (ii) induction of Id-specific T cells and anti-Id antibodies in therapy of multiple myeloma and Id^+^B lymphomas. However, immunogenicity of Ids is generally considered to be poor. The need for improving the immunogenicity of Id-immunization is underscored by two negative phase III trials on Id-vaccination of B lymphoma patients (Levy et al., 2008; Freedman et al., 2009), while a third phase III trial showed a marginal effect (Schuster et al., 2011). These trials employed vaccination with Id-keyhole limpet hemocyanin (KLH) conjugates and adjuvants.

The low immunogenicity of Id is probably related to B and T cell tolerance, the levels of which may vary with various individual Ids. As long as the sole object of Id-vaccination is to obtain anti-Id antibodies, and assuming that anti-Id B cells usually are not tolerant, the problem of T cell tolerance can be circumvented by conjugation of the Ig to a powerful carrier molecule, such as KLH, that contain a multitude of T cell epitopes that should fit polymorphic MHC II molecules of most individuals (Kaminski et al., 1987). Thus, Ig-KLH conjugates have become a “gold standard” for Id-vaccination of B lymphoma patients (Bendandi et al., 1999; Inoges et al., 2006; Redfern et al., 2006; Levy et al., 2008; Freedman et al., 2009; Timmerman et al., 2009; Schuster et al., 2011). However, the “carrier strategy” does indeed not solve the problem of lack of Id-specific T cell help – it just circumvents it. Ig-KLH is commonly delivered with adjuvants that increase the levels of anti-Id antibody responses. The empirical science of adjuvants has developed considerably during the last decade, and recent progress in the field of receptors of innate immunity, such as Toll-like receptors (TLRs), is likely to generate more powerful and clinically acceptable adjuvants. Given the poor effect of Id-KLH vaccinations in phase III trials, immunization strategies that elicit potent Id-specific T cells, and not only anti-Id antibodies, might be desirable. Apart from the low efficacy, the Id-KLH strategy for Id-vaccination is labor intensive. Id-KLH vaccination has for the most part been applied to patients with follicular B cell lymphomas. These lymphomas arise from germinal center B cells, and their B cell receptors (BCR) for antigen are usually marked by somatic mutations. Hence, lymphoma BCR of different patients are different, and Id-vaccines have to be prepared for each individual patient. Traditionally, this has involved fusion between lymphoma cells and non-secreting myeloma cells (rescue fusion) to obtain soluble lymphoma Ig for conjugation with KLH (Schuster et al., 2011). More recently, recombinant technologies have been applied to obtain lymphoma V region DNA sequences, and to express these as Ig used for preparation of Ig-KLH conjugates (Levy et al., 2008; Freedman et al., 2009). These strategies for generation of individual patient-dedicated protein Id-KLH vaccines are prohibitively time-consuming and costly.

There are a number of more recent innovative approaches to improve Id-vaccination. In one approach, Id-specific immune responses in mice could be enhanced by DNA vaccination with a single-chain fragment variable (scFv)-bacterial antigen (fragment C of tetanus toxin) fusion (King et al., 1998). In another approach, Id was fused with lysosomal-associated membrane protein 1 (Id-LAMP1), and integrated in recombinant vaccinia virus (rVV). Dendritic cells (DCs) infected *in vitro* with Id-LAMP1 rVV were used for immunization of mice, resulting in Id-specific T cell responses and tumor protection (Muraro et al., 2005). In an APC-targeting approach, but using protein rather than DNA, Id^+^ scFv was fused with scFv specific for CD19 in a diabody format. Targeting of CD19 on B cells increased Id-specific responses (Ng et al., 2012). Finally, B lymphoma cells were generated that by gene targeting had their endogenous heavy (H) chain replaced by a human H chain. Such engineered lymphoma cells were used to immunize mice, and induced a T cell-mediated protection against wild-type B cell lymphoma (Selmayr et al., 2000). These studies have contributed interesting approaches for Id-immunization, but will not be discussed further as they are not examples of APC-targeted DNA Id-vaccines, which is the theme of the present paper.

In this review, it is considered that a combination of three elements could enhance Id-vaccination: (i) genetic construction of patient-specific Id-vaccines, (ii) targeting of these to APC, and (iii) delivery as DNA. Such a strategy could reduce the cost of preparing individual vaccines and improve anti-Id responses, particularly Id-specific T cell responses. Of these three elements, genetic construction of Id-vaccines, as well as delivery of Id-vaccines as DNA, was already reported in the nineties (Hawkins et al., 1993; Stevenson et al., 1995; Syrengelas et al., 1996; King et al., 1998). APC-targeted DNA Id-vaccines is more recent (Biragyn et al., 1999; Ruffini et al., 2004, 2010; Fredriksen et al., 2006; Fredriksen and Bogen, 2007; Schjetne et al., 2007; Qin et al., 2009;Froyland et al., 2011), and is the focus of the text to follow.

## TARGETING ANTIGEN TO ANTIGEN-PRESENTING CELLS INCREASES IMMUNE RESPONSES

Given the poor immunogenicity and labor-intensive production of Id-vaccines, new vaccination strategies are warranted. It has been known since the eighties that targeting of antigen to APC increases both T and B cell responses (Kawamura and Berzofsky, 1986; Carayanniotis and Barber, 1987; Casten and Pierce, 1988; Baiu et al., 1999). These pioneering studies were done by chemical conjugation of antigen to antibodies specific for surface molecules such as BCR, MHC II, FcR, and complement receptors (Kawamura and Berzofsky, 1986; Carayanniotis and Barber, 1987; Baiu et al., 1999) on APC. However, chemical conjugation often results in different Ag:Ig ratios, therefore, chemical conjugates are fraught with batch to batch variation. This problem is solved by genetic fusion of antigen to APC-specific Ab, ensuring a defined fusion protein, as done by the authors and others in the late nineties (Biragyn et al., 1999; Lunde et al., 1999, 2002). This recombinant Ig strategy for APC has become very popular, e.g., in work targeting surface molecules on DCs such as DEC205 (Hawiger et al., 2001; Demangel et al., 2005; Kretschmer et al., 2006) and Clec9a (Lahoud et al., 2011).

## APC-TARGETING OF T CELL EPITOPES INSERTED INTO THE IMMUNOGLOBULIN STRUCTURE

Together with Sandlie, Lunde and Bogen developed a recombinant Ig-based strategy for APC-targeting (Lunde et al., 1999). This strategy was based on the observation, described above, that Ig are endocytosed and processed by APC, and that CDR3 Id-peptides are displayed on MHC class II molecules for recognition by Id-specific CD4^+^T cells (Bogen et al., 1986b; Weiss and Bogen, 1991). Thus, if a CDR3 epitope could be excised from the Ig molecule by the antigen processing machinery, T cell epitopes engineered to replace loops between β–strands in constant (C) domains of Ig molecules should also be excised for MHC-presentation. This proved to be the case (Lunde et al., 1997). Later work demonstrated that many loops throughout the C-region, especially in the C_H_2 domain, are suited for T cell epitope replacement (Flobakk et al., 2008). Indeed, multiple substitutions can be made within a single Ig molecule, suggesting the possibility of using recombinant Ig for multivaccine purposes (Rasmussen et al., 2012).

Importantly, since the T cell epitopes were inserted into the C-domains, the original V regions should be dispensable and therefore exchangeable with APC-specific V regions cloned from B cell hybridomas of appropriate specificity. Such dual-substituted recombinant Ig should target T cell epitopes to APC, resulting in improved T cell responses. To test this idea, V regions specific for IgD (Lunde et al., 1999; Rasmussen et al., 2001), MHC II (Lunde et al., 2002), and CD40 (Schjetne et al., 2007) were exchanged with original V regions, and T cell epitopes were inserted into C domain loops. Such APC-targeted recombinant Ig molecules, called Troybodies, had an enhanced ability (×10^2^–10^4^) to stimulate CD4^+^T cell specific for model T cell epitopes (Lunde et al., 1999, 2002; Rasmussen et al., 2001;Schjetne et al., 2007). A similar strategy was later used by Hawiger et al. (2001), who genetically attached antigen to the C-terminus of heavy chains of DC-specific anti-DEC205 mAb. This strategy has become popular and has been used with minor variations in a large number of studies.

## TARGETING OF Id IN A scFv FORMAT TO ANTIGEN-PRESENTING CELLS

In 1996/1997, Bjarne Bogen had a sabbatical in the lab of Ron Levy at Stanford University. This experience made it evident that efficient induction of anti-idiotypic antibodies was important for immunotherapy of B lymphoma cells. In this respect, Troybodies (see above) were deficient because short Id-sequences introduced into the Ig structure, although stimulatory for T cells, did not generate conformational Id determinants recognized by B cells. Another problem of Troybodies was that short Id-sequences only would be presented by certain MHC molecules present in only a fraction of individuals in the population.

A solution to these two problems seemed to be to include patients’ B lymphoma V regions into an APC-targeted vaccine molecule. This should allow induction of anti-idiotypic antibodies and possibly also induction of Id-specific T cells. Such an APC-targeted idiotypic vaccine should be bipolar, with APC specificity oriented in one direction and idiotypic V regions in the other direction. Moreover, similar to IgG antibodies, the molecule should be bivalent in order to increase avidity for APC. Bivalency for idiotypic antigen should also be beneficial, since bivalency would engender cross linking of BCR of anti-Id B cells, probably inducing better anti-Id antibody responses. Finally, the molecule should be devoid of Fc-associated biological effector functions such as binding to Fc receptors and complement activation. This statement may appear surprising since binding to Fc and complement receptors could indeed result in a positive outcome (Fearon and Locksley, 1996). However, binding to such receptors could potentially result in absorption of vaccine molecules and thereby deviation from intended, optimal APC targets, potentially blurring the results.

## HOMODIMERIC VACCINE MOLECULES THAT BIVALENTLY TARGET DIMERIC IDIOTYPIC ANTIGEN TO APC

**FIGURE 1:**
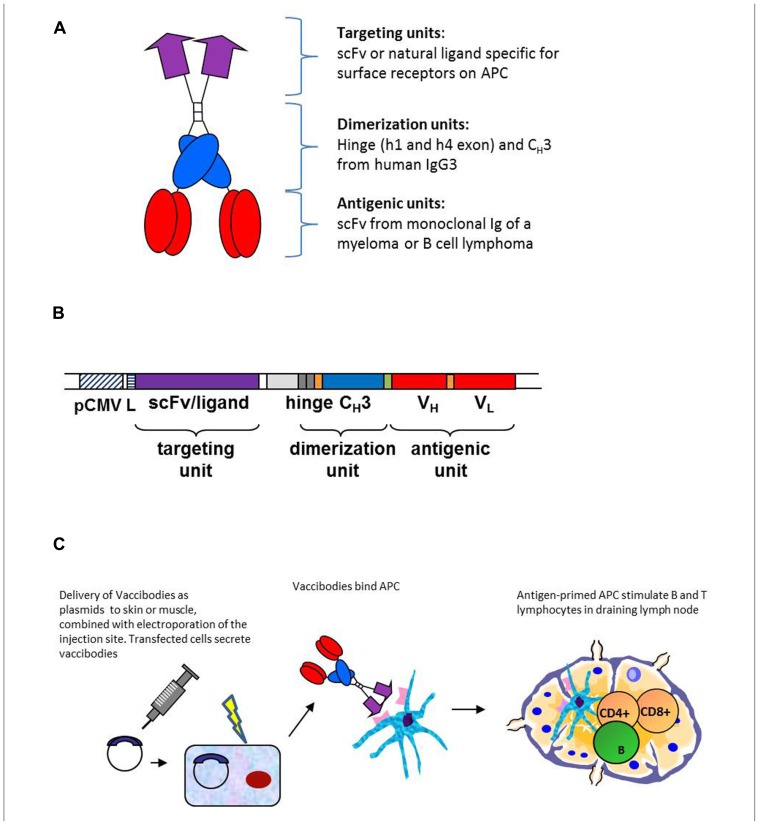
**Design, construction, and DNA delivery of APC-targeted vaccines**. **(A)** The vaccine proteins are heterodimers. Each chain is composed of an N-terminal targeting unit [scFv(V_L_ + V_H_) or chemokines, *violet*], that bind surface receptors on APC, a dimerization unit composed of a shortened human Ig hinge and C_H_3 domain (*blue*), and an antigenic unit corresponding to tumor-specific scFv from a B cell tumor (multiple myeloma, B cell lymphoma; *red*). As non-targeted controls we used vaccine molecule versions where the targeting unit was replaced with either scFv specific for the hapten NIP, or inactive (mutated) chemokine. **(B)** Gene construct. The targeting unit is inserted into the V cassette of the pLNOH2 vector. The dimerization unit, composed of h1 and h4 hinge exons and the C_H_3 exon of human IgG3 (*blue*), is linked to the antigenic unit (*red*) and inserted together in the C cassette of pLNOH2. The (G_4_S)_3_ linkers (*orange*) and the GLSGL linker (*green*) are indicated. The gene is expressed from a CMV promoter (hatched) and a leader sequence (*striated*) of the pLNOH2 vector (*uncolored*). Upstream of h1 is an intronic sequence (*light* *gray*) Reproduced with modifications from *Mol. Ther.* 13: 776–785, 2006, with permission from the publisher. **(C)** Transfected cells secrete vaccine proteins that target antigen presenting cells. The APC travels to the draining lymph node where it meets CD4^+^ T cells, B cells and CD8^+^ T cells.

Based on the deliberations made above, the APC-targeted Id-vaccine molecule was constructed as a homodimer, each chain of the homodimer being composed of the following units: a targeting unit specific for APC, a homodimerization unit and Id antigen expressed as scFv (**Figure 1A**). These units were connected by short linkers (**Figure 1B**). The molecules were genetically constructed by use of a shuttle vector where the targeting units, the dimerization unit and the Id scFv could be easily exchanged (**Figure 1B**). An overview of published molecules used for Id-vaccination is given in **Table 1**.

**Table 1:** TABLE 1. Efficiency of various targeting units in APC-targeted Id-vaccines*.

	Mouse αMHC class II Fredriksen et al., 2006; Froyland et al., 2011)	Mouse αCD40 (Schjetne et al., 2007)	Mouse MIP-1α (Fredriksen and Bogen, 2007)¤	Human MIP-1α (Ruffini et al., 2010)	Mouse RANTES (Fredriksen and Bogen, 2007)¤
CD4^+^ T cell responses in vitro	++	++ (+)	+++	++	++(+)
CD4^+^ T cell responses in vitro	+++	++	+++	n.t.	++
Anti-Id antibody responses	+++	++	++	++	+
Protection against Id^+^ tumors	60%	50–60%	70–80%^#^	n.t.	40–50%

**FIGURE 2:**
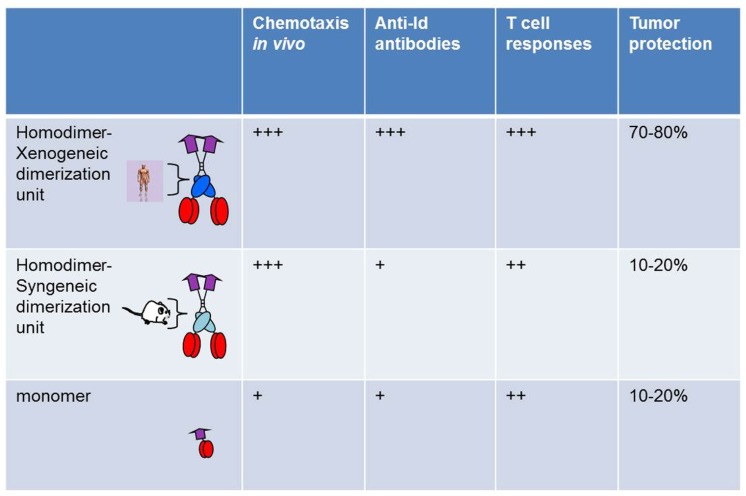
**Bivalency and xenogeneic sequences increase immunogenicity of vaccine molecules**. Homodimers with human xenogeneic dimerization motifs were compared with homodimers with murine syngeneic dimerization motifs and monomers for induction of chemotaxis, Id-specific T and B cell responses, and protection against Id^+^ tumor challenge. Vaccine molecules had MIP1-α as targeting unit and scFv^315^ or scFv^A20^ as antigenic unit (Fredriksen and Bogen, 2007).

* Responses are qualitatively evaluated +++, ++, + compared to non-targeted controls. Since side by side comparisons were not done, and results were published separately (except MIP-1α and RANTES), exact comparison is impossible. The indicated refs should be consulted for details. Id-specific responses were mostly tested with scFv from the MOPC315 mouse myeloma, but also with scFv from the mouse A20 B lymphoma. In one study (Froyland et al., 2011) humanMMscFv from 4 patients were tested. The results refer to vaccine molecules with a human dimerization motif (shortened hinge + C_H_3). For the reference marked with ¤ besides the ref, equivalent mouse dimerization motif was tested in parallel (see also **Figure 2**).

# Antibody depletion experiments indicated that CD8^+^ T cells conferred most of the protection against MOPC315 MM cells. n.t., not tested.

As for targeting units, the following have been published: anti-mouse MHC class II as scFv (Fredriksen et al., 2006; Froyland et al., 2011), agonistic anti-mouse CD40 as scFv (Schjetne et al., 2007), mouse MIP1α (CCL3) and RANTES (CCL5) binding CCR1,3,5 on APC (Fredriksen and Bogen, 2007), and human MIP1α (LD78β; Ruffini et al., 2010). For all these targeting units, negative controls not binding APC have been constructed, such as scFv specific for the hapten NIP (Fredriksen et al., 2006; Schjetne et al., 2007; Froyland et al., 2011), or chemokine versions where the structural integrity has been destroyed by introduction of a C11A mutation that disrupts a disulfide bond (Fredriksen and Bogen, 2007; Ruffini et al., 2010). The scFv targeting units have been expressed in the V_H-VL_ order with maintenance of specificity (an exception has been anti-TLR2, used in other vaccine molecule studies not reviewed here, where reversal of orientation to V_L_-V_H_ improved binding to TLR2; Tunheim et al., 2007). The different scFv used for targeting appeared to influence the level of secretion of fusion protein, in the order αNIP > αMHCII > αCD40. Concerning chemokines as targeting moieties, different chemokines have been expressed approximately to the same levels in secreted homodimeric fusion proteins, and maintained their binding characteristics and chemotactic properties.

As for homodimerization motif, we have used a shortened human γ3 Ig hinge with cysteines available for disulfide bond formation. The shortened hinge has been connected via a G_3_S_2_G_3_SG linker to human (γ3) or mouse (γ2b) C_H_3 domains that associate non-covalently. Thus, the shortened hinge and the C_H_3 domain should confer homodimerization. Covalent dimerization of proteins secreted by transfected HEK293 cells has been partial, as estimated by SDS-PAGE under reducing or non-reducing conditions, varying roughly from close to 100% homodimerization to about 50%. Degree of covalent dimerization appears to be influenced by targeting and antigenic units, but there are also other factors involved. The degree of non-covalent homodimerization under physiological conditions has not yet been tested.

As for idiotypic antigens we have used scFv^315^ (from the BALB/c mouse plasmacytoma MOPC315; Eisen et al., 1968), scFv^A20^ (from the BALB/c B cell lymphoma A20; Kim et al., 1979), and scFv of four human multiple myeloma patients (Froyland et al., 2011). ScFv have been in the V_H-VL_ order, connected by a (G_4_S)^3^ linker. In general, we have experienced few if any problems in expressing these various Id scFv as part of homodimeric vaccine proteins secreted by transiently transfected HEK293 cells. Id scFv appeared to fold correctly in the vaccine molecule format since they bound anti-Id mAb (Fredriksen et al., 2006; Fredriksen and Bogen, 2007; Schjetne et al., 2007; Ruffini et al., 2010; Froyland et al., 2011).

## TARGETED IDIOTYPIC VACCINE, TESTED AS PROTEINS, ENHANCE T AND B CELL RESPONSES

Both anti-MHCII-scFv^315^ and MIP-1α–scFv^315^ proteins were about ×1,000-fold more effective, on a molar basis, at stimulating Id-specific CD4^+^T cells from Id^315^-specific TCR-transgenic mice (Fredriksen et al., 2006; Fredriksen and Bogen, 2007). These results are highly promising for *in vivo* immunization with proteins. Unfortunately, it has until now been cumbersome to produce sufficient amounts of purified vaccine proteins from transiently transfected HEK293 and stably transfected NS0 cells for extensive immunization experiments. However, a single injection of 100 µg of MIP-1α–scFv^315^ vaccine proteins in PBS induced a 20% protection against a challenge with MOPC315 plasmacytoma cells on day 14 after immunization, compared to nil protection obtained with the non-targeted control (Fredriksen and Bogen, 2007). These results are encouraging, but more efficient protein production is needed for extensive investigations on protein vaccination to be carried out.

## DELIVERY OF APC-TARGETED Id AS DNA VACCINES INDUCE STRONG ANTI-Id RESPONSES AND TUMOR PROTECTION

Given the problems in producing sufficient proteins for immunization, we resorted to perform DNA immunization. The rationale for this choice was the previous finding that Ig H and L chain genes, when injected intramuscularly, resulted in prolonged production of assembled and functional H + L mAb molecules that could be detected in serum (Tjelle et al., 2004). Production of mAb by muscle cells was dependent upon electroporation of the injection site, which enhances the number of DNA-transfected cells (Mathiesen, 1999), and thus protein production and secretion. The particular mAb produced and secreted by muscle, anti-MHC II (I-E^d^) could be detected in serum of a mouse that lacked I-E^d^ but not in a mouse that expressed I-E^d^. Thus, muscle-produced mAb bound MHC II molecules *in vivo *(Tjelle et al., 2004)*.*

On this basis, we considered it possible that injection of homodimeric vaccine plasmid, combined with electroporation, could result in secretion of vaccine fusion protein into extracellular fluid, followed by binding to surface molecules on APC. Experiments to test this were first done with anti-MHCII-scFv^315^ plasmid, using anti-NIP-scFv^315^ as non-targeted control (Fredriksen et al., 2006). The results demonstrated that electroporation was needed to detect vaccine proteins in serum. Moreover, anti-MHC II (I-E^d^) vaccine proteins were detected in mice lacking I-E^d^ but were absent in mice expressing I-E^d^, consistent with absorption on MHC II^+^APC. These results led to a model for how targeted DNA vaccines work. Briefly, transfected cells secrete vaccine protein that target APC, followed by drainage to lymph nodes for initiation of T and B cell responses (**Figure 1C**). The distinction from conventional DNA immunization, where the transfected cells themselves are thought to serve as APC, is evident.

Next, induction of anti-Id antibodies in serum was followed (Fredriksen et al., 2006). anti-MHC II-scFv^315^ induced a strong and rapid anti-idiotypic antibody response compared to non-targeted (anti-NIP) control. One immunization was sufficient for detection of anti-Id antibodies within 14 days. antibody levels increased to day 60, then declined until day 170. Three immunizations spaced 21 days apart, increased antibody levels. A DNA dose-sparing effect of targeting was observed. These results were encouraging since M315 protein is poorly immunogenic and prolonged immunization schedules have been required to elicit anti-Id antibody responses.

Concerning *in vivo* T cell responses, MHC II-targeted DNA immunization was 100–1,000 times better at stimulation of Id-specific CD4^+^ T cells, as revealed by immunizing TCR-transgenic mice and BrdU incorporation experiments (Fredriksen et al., 2006).

As for resistance to tumor challenge, mice immunized once with anti-MHC II-scFv^315^ resisted a challenge with MOPC315 tumor cells while control mice immunized with non-targeted control were not protected. Similar results were obtained with DNA vaccines constructed for BALB/c B lymphoma A20, where vaccination also induced tumor resistance.

## EXTENSION TO OTHER TARGETING UNITS

Two other mouse targeting units have been published: agonistic scFv^α^^CD40^ based on the agonistic anti-CD40 mAb FGK45 (Schjetne et al., 2007) and the mouse chemokines MIP-1α and RANTES (Fredriksen and Bogen, 2007). The former was indeed selected because agonistic targeting of CD40 could serve two functions: (i) activation of APC and (ii) loading with Id-peptide. Both effects were shown to be induced by vaccine molecules (Schjetne et al., 2007). Immunization with scFv^α^^CD40^–scFv^315^DNA vaccines induced anti-Id antibodies and protection against tumor challenge.

MIP-1α and RANTES were selected because they are inflammatory chemokines and because previous experiments performed in our laboratory in surrogate systems employing mAbs to chemokine receptors had indicated that CCR1, 3, and 5 (to which MIP-1α and RANTES bind) could be promising targets (Schjetne et al., 2003). Moreover, chemotactic activity could cause APC to accumulate at site of injection, resulting in enhanced vaccine uptake and presentation. MIP-1α performed better than RANTES in assays for chemotaxis, induction of anti-Id antibodies and tumor protection. These results of the efficacy of the various targeting units are summarized in Table 1.

It should be emphasized that genetic fusion of other chemokines (MIP-3α and MCP3) to scFv^Id^ has previously been successfully used as DNA vaccines by Biragyn and Kwak in A20, 38C13, and MOPC315 mouse tumor models (Biragyn et al., 1999; Ruffini et al., 2004; Qin et al., 2009; see below). In these studies, the fusion proteins had a monomeric form where the chemokine moiety was directly attached to scFv. Interestingly, when the chemokine-scFv DNA vaccine was combined with myotoxins that induce sterile inflammation with recruitment of APC at the intramuscular injection site, enhanced antitumor immunity was observed (Qin et al., 2009).

## BIVALENCY AND XENOGENEIC SEQUENCES INCREASE IMMUNOGENICITY OF TARGETED BIVALENT IDIOTYPIC VACCINES

A comparison was done between bivalent and monovalent Id fusion protein with MIP-1α as targeting unit. The monovalent form was constructed *ad modum* Biragyn and Kwak, where the chemokine is directly attached to idiotypic scFv (Biragyn et al., 1999). Compared on a molar basis, the bivalent form had a higher chemotactic activity both *in vitro* and *in vivo*, was more efficient at stimulation of T cells *in vitro* and *in vivo*, had an increased ability to induce anti-Id antibodies, and induced a higher resistance to tumor challenge. Thus, bivalency appeared to increase efficacy of the vaccine molecule in a variety of short-term and long-term assays *in vitro* and *in vivo *(**Figure 2**). A major part of the enhanced efficiency is likely to be due to bivalency of the targeting unit MIP-1α, and hence enhanced chemotactic activity and binding to APC.

A side by side comparison of vaccine molecules having human (γ3) or mouse (γ2b) C_H_3 domains was undertaken, using MIP-1α-targeted vaccine molecules. While no influence was seen in short-term assays, such as chemotaxis or stimulation of Id-specific T cells *in vitro* or *in vivo*, a clear influence was observed in long-term *in vivo* assays such as induction of antibodies and resistance to an Id^+^ tumor challenge (Fredriksen and Bogen, 2007; **Figure 2**). Thus, xenogeneic sequences in the homodimerization domain appeared to increase immunogenicity. However, vaccine constructs with mouse γ2b homodimerization domain appear to be less well secreted by transfected cells *in vitro*. If this is also the case upon DNA vaccination and electroporation, i.e., that transfected cells *in vivo* produce less vaccine protein, this could have contributed to the decreased immunogenicity of mouse γ2b-containing DNA vaccines. A possible explanation for these findings could be that xenogeneic C_H_3 sequences are presented on MHC class II molecules. Xenogeneic sequences might be required for generation of sufficient help, since the T cell repertoire for syngeneic V regions is purged of T cells responding to germline–encoded sequences and is limited to recognition of Id-peptides expressing somatic mutations or V(D)J junctional sequences (Bogen et al., 1985, 1986a,b, 1993; Eyerman and Wysocki, 1994; Eyerman et al., 1996) reviewed in Bogen and Ruffini (2009). This explanation is consistent with the contribution of KLH to immunogenicity of Id in Id-KLH conjugates, by induction of KLH-specific T helper cells. Based on such results one may envisage that deliberate insertion of foreign promiscuous T cell epitopes, with ability to bind most MHC molecules in the species, could increase efficiency of the targeted vaccine molecules.

## TARGETING SPECIFICITY INFLUENCES PHENOTYPE OF ELICITED IMMUNE RESPONSES

It is clearly of great importance to be able to direct the type of anti-Id immune responses elicited by Id-vaccination. For example, in B cell lymphoma, anti-Id antibodies appear to be important for tumor eradication (Syrengelas and Levy, 1999), while in multiple myeloma, Id-specific T cells seems to be the therapeutically most important arm of Id-immunity (Lauritzsen et al., 1994; Dembic et al., 2000; Corthay et al., 2005; Haabeth et al., 2011). Thus, Id-vaccines should elicit the kind of immune response suitable for the particular B cell tumor disease of the patient. Steering the phenotype of Id-immunity in the desired direction might be obtained by varying the targeting units of the bivalent idiotypic vaccine molecule. This has not yet been investigated fully, but available data suggest that targeting of Id-vaccines with scFv^α MHC class II^ induces high amounts of antibodies while targeting with MIP-1α induces more T cells (Fredriksen et al., 2006; Fredriksen and Bogen, 2007). It might be of particular merit to target particular subsets of APC, i.e., CD8^+^DCs, the latter being known for their ability to cross present antigen to cytotoxic CD8 T cells.

## A HYPOTHETICAL MODEL FOR ACTION OF APC-TARGETED BIVALENT VACCINE MOLECULES

**FIGURE 3:**
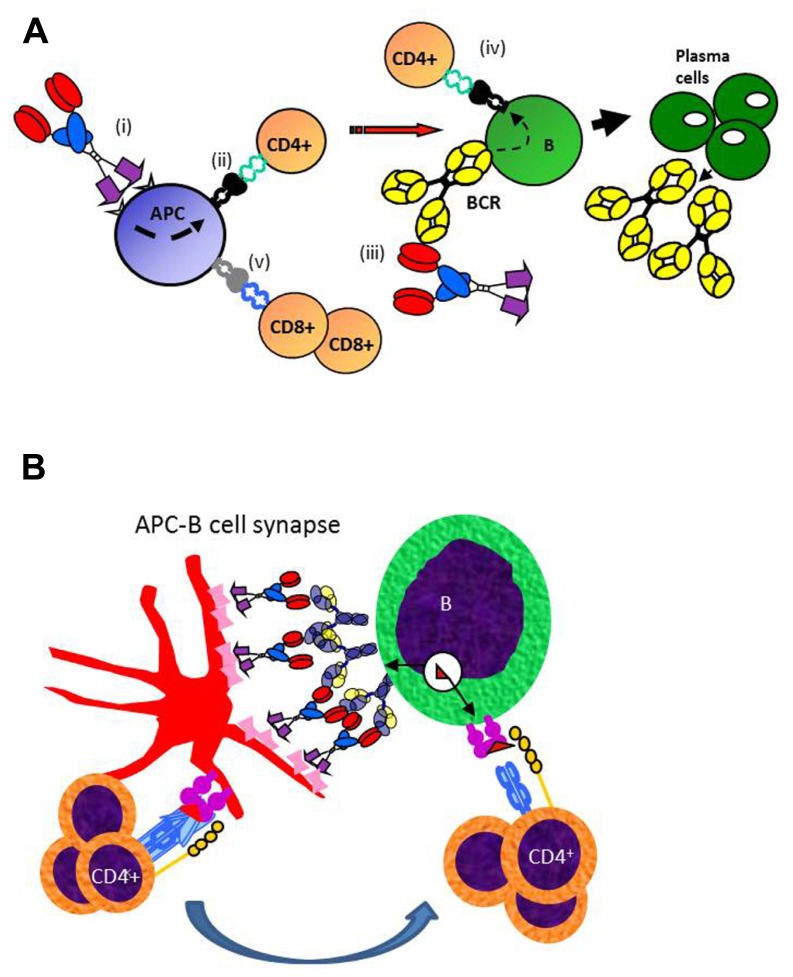
**Proposed mechanism for how APC-targeted homodimeric vaccine molecules efficiently stimulate both T and B cell responses**. **(A)** (i) Vaccibodies bind to surface molecules on APC, such as dendritic cells and induce their maturation. (ii) Vaccibodies are processed and peptides from the antigenic unit are presented on MHC class II molecules of APC to naïve CD4^+^ T cells that become effector T cells. (iii) B cells with a B cell receptor (BCR)-specific for conformational determinants on complete vaccibodies internalize vaccibodies; process them, and present antigenic peptides on their MHC class II molecules to the effector CD4^+^ T cells. (iv) B cells receive help from the effector CD4^+^ T cells and develop into plasma cells that secrete antibodies. Finally, antigen-specific antibodies and CD4^+^ T cells cause the elimination of the antigen. (v) Vaccibodies with certain targeting units (MIP-1α) can induce presentation on MHC class I, and cross-priming of CD8^+^ T cells is thought to be particularly important for viral infections and cancer. **(B)** A variant model where homodimeric vaccine molecules bridge APC and B cells, resulting in a “tug of war” for antigen. T cell could interact with MHC II-Ag complexes on both APC and B cells, as indicated.

The mechanism for why targeted vaccine molecules (called vaccibodies) improve T and B cell responses to Id is hypothesized in **Figure 3**. Briefly, targeting of vaccine molecules to APC such as DCs should result in efficient stimulation of CD4^+^ T cells. Simultaneously, B cells should bind conformational determinants on the antigenic units, and process and present the antigen on their MHC class II molecules. Primed B cells will receive help from the CD4^+^ T cells already stimulated by DCs, resulting in generation of plasma cells and antibody production. In addition, certain targeting units, such as MIP-1α, may by unknown mechanisms result in cross-presentation of Id on MHC class I molecules and generation of CD8^+^ T cells (Fredriksen and Bogen, 2007; **Figure 3A**). A variant model may be considered where the vaccine molecules could bridge DCs and B cells, as in an APC B cell synapse (Batista et al., 2001). A “tug of war” between the APC and the B cell could result in Ag being displayed as peptides on MHC II molecules of both cells (**Figure 3B**). CD4^+^ T cells could interact with both the APC and the B cell, either consecutively or simultaneously, in the latter case forming a three-member cellular complex.

## EXTENSION TO HUMAN TARGETING UNITS AND HUMAN IDIOTYPES FROM PATIENTS

Homologous chemokines are expressed in mouse and man and indeed most mammal species. Therefore, since mouse MIP-1α gave encouraging results in mice (Fredriksen and Bogen, 2007), it was of particular interest to test if human MIP-1α could also function as a targeting unit. Moreover, if human MIP-1α bound to mouse chemokine receptors, a vaccine intended for human use could first be tested in mice prior to human application. Such studies could pave the way for application of APC-targeted Id-vaccines in humans.

In humans, there are two homologs of MIP-1α, LD78α and LD78β both sharing 74% homology with the mouse MIP-1α. The two variants are 96% homologous, however, while LD78α-targeted vaccine was unable to bind strongly to both murine and human chemokine receptors, LD78β-fusion vaccines bind both murine, human and macaque chemokine receptors. Furthermore, LD78β-targeted vaccines demonstrated increased ability to activate CD4^+^ T cells and antigen-specific antibodies in mice models (Ruffini et al., 2010). Thus, the human vaccine product can be tested for functionality in both murine and non-human primate models before entering the clinic.

The overall aim of the studies reviewed herein is to develop Id-vaccines that work in patients. We therefore genetically constructed scFv from multiple myeloma cells obtained from bone marrow of four patients, and inserted these into vaccine molecules specific for mouse MHC II (I-E^d^; Fredriksen et al., 2006). Mice DNA-immunized with these constructs produced antibodies that in ELISA bound the particular purified serum myeloma protein corresponding to the scFv used, but poorly or not at all to myeloma proteins from the other three patients. By this criterium, the scFv must correctly fold when produced and secreted by transfected mouse cells. anti-idiotypic antibody titers were much higher in mice immunized with MHC II-targeted vaccine constructs compared with the non-targeted (NIP-specific) control (Fredriksen et al., 2006). The anti-idiotypic antibodies could be used to establish ELISAs specific for the myeloma protein of each patient (Froyland et al., 2011). These ELISAs were about 100-fold more sensitive than standard immunofixation for detection of myeloma protein in serum, and could be used for early detection of recurrence of disease. 

## FUTURE PERSPECTIVES: EXTENSION TO VACCINATION OF HUMANS WITH B CELL CANCERS

As reviewed above, scFv can be constructed from patients with B cell malignancies, and inserted into targeted bivalent DNA vaccines (Froyland et al., 2011). The ease and rapidity with which the vaccines can be genetically constructed and produced as DNA are clear advantages for generation of individual patient-specific Id-vaccines. Mice immunized with MHC II-targeted constructs make anti-Id antibodies to human Id after a single DNA injection combined with electroporation (Froyland et al., 2011).

In future work, we plan to equip patient-specific scFv DNA vaccines with targeting units specific for human APC. Provided the targeting unit cross-react between human and mouse APC, which is the case for human MIP-1α (LD78β; see above), the DNA vaccine intended for human use can be tested in mice prior to human application. It should be noted that DNA vaccination with electroporation has been performed in humans without serious side effects (van Drunen Littel-van den Hurk and Hannaman, 2010; Vasan et al., 2011). However, the DNA vaccines tested so far have been of a non-targeted nature. In our long-term experiments in mice, we have yet to observe any detrimental side effects of targeted DNA vaccines.

## Conflict of Interest Statement

The authors are inventors on several patent applications, submitted via their employers (University of Oslo, Oslo University Hospital), related to the described vaccine concept (vaccibodies).Agnete Fredriksen is CSO and Bjarne Bogen is head of the scientific panel of Vaccibody AS. All three authors have shares in the company.
